# Evolutionary dynamics of rRNA gene clusters in cichlid fish

**DOI:** 10.1186/1471-2148-12-198

**Published:** 2012-10-05

**Authors:** Rafael T Nakajima, Diogo C Cabral-de-Mello, Guilherme T Valente, Paulo C Venere, Cesar Martins

**Affiliations:** 1 Bioscience Institute, Morphology Department, UNESP - Sao Paulo State University, Botucatu, SP, 18618-970, Brazil; 2 Bioscience Institute, Biology Department, UNESP - Sao Paulo State University, Rio Claro, SP, Brazil; 3 Institute of Biological Science and Health, UFMT - Federal University of Mato Grosso, Pontal do Araguaia, MT, Brazil

## Abstract

**Background:**

Among multigene families, ribosomal RNA (rRNA) genes are the most frequently studied and have been explored as cytogenetic markers to study the evolutionary history of karyotypes among animals and plants. In this report, we applied cytogenetic and genomic methods to investigate the organization of rRNA genes among cichlid fishes. Cichlids are a group of fishes that are of increasing scientific interest due to their rapid and convergent adaptive radiation, which has led to extensive ecological diversity.

**Results:**

The present paper reports the cytogenetic mapping of the 5S rRNA genes from 18 South American, 22 African and one Asian species and the 18S rRNA genes from 3 African species. The data obtained were comparatively analyzed with previously published information related to the mapping of rRNA genes in cichlids. The number of 5S rRNA clusters per diploid genome ranged from 2 to 15, with the most common pattern being the presence of 2 chromosomes bearing a 5S rDNA cluster. Regarding 18S rDNA mapping, the number of sites ranged from 2 to 6, with the most common pattern being the presence of 2 sites per diploid genome. Furthermore, searching the *Oreochromis niloticus* genome database led to the identification of a total of 59 copies of 5S rRNA and 38 copies of 18S rRNA genes that were distributed in several genomic scaffolds. The rRNA genes were frequently flanked by transposable elements (TEs) and spread throughout the genome, complementing the FISH analysis that detect only clustered copies of rRNA genes.

**Conclusions:**

The organization of rRNA gene clusters seems to reflect their intense and particular evolutionary pathway and not the evolutionary history of the associated taxa. The possible role of TEs as one source of rRNA gene movement, that could generates the spreading of ribosomal clusters/copies, is discussed. The present paper reinforces the notion that the integration of cytogenetic data and genomic analysis provides a more complete picture for understanding the organization of repeated sequences in the genome.

## Background

Ribosomal RNA (rRNA) multigene families are organized into two distinct classes that are tandemly arrayed in eukaryotic genomes. The major class is formed by an external transcribed spacer followed by the transcribing regions of the 18S, 5.8S and 25S/28S rRNAs, which are separated from each other by two internal transcribed spacers (ITS), ITS1 and ITS2. The minor class (5S rRNA genes) consists of multiple copies of a highly conserved 120 bp transcribing region that is separated by a variable non-transcribed region (NTS) [1,2]. These sequences are characterized by a flexible organization at both the (i) chromosomal level, varying in both number and position in the karyotypes even within species (for example, [3-5]), and (ii) the nucleotide sequence level, with differences occurring mostly in the spacers of 45S and 5S rDNA [1,2,6]. Moreover, some cases of co-localization/interspersion between these two multigene families have been reported, in addition to their association with other multigene families such as histones and small nuclear RNA (snRNA) genes [7-13].

With more than 3,000 living species, Cichlidae is one of the most species-rich families of Perciformes. This group is separated into four monophyletic lineages: Etroplinae (Indians), Ptychrominae (Malagasy), Cichlinae (Neotropicals) and Pseudocrenilabrinae (Africans). The evolutionary history of cichlids is in accordance with the fragmentation of the Gondwana landmass [14]. Cichlids from Madagascar and India constitute the most basal group to diverge from ancestral African–Neotropical cichlids, and their divergence coincides with the separation of the India-Madagascar subcontinent close to 150 million years ago (MYA) [15]. More recently (approximately 65 MYA), Neotropical and African cichlids diverged following the separation of Africa and South America [16]. Cichlinae and Pseudocrenilabrinae contain the majority of the described species. Cichlinae representatives are divided into seven tribes: Cichlini, Retroculini, Astronotini, Chaetobranchini, Geophagini, Cichlasomatini and Heroini. Pseudocrenilabrine is composed of three major groups that are not recognized as valid taxonomic units, pelmatochromine, haplochromine and tilapiine, with great diversity in the Great Lakes of East Africa, Tanganyika, Malawi and Victoria [17-21]. Among vertebrates, cichlids, especially the African representatives, have been used as models to study evolutionary mechanisms due to their rapid and convergent evolutionary radiation [20,22,23].

To date, 135 species of cichlids have been cytogenetically analyzed, the diploid number of which is predominantly 2n = 48 (more than 60% of the studied species), although variations ranging from 2n = 38 to 2n = 60 have been described. For African species, the modal diploid number is 2n = 44, and in Neotropical cichlids, the most common chromosome number is 2n = 48, which is considered to be the ancestral characteristic for this family [24]. The understanding of cichlid karyotypes has advanced after fluorescence *in situ* hybridization (FISH) technology was applied to the chromosomal physical mapping (cytogenetic mapping or FISH mapping) of DNA sequences. FISH mapping identifies useful chromosomal markers that can be applied to studies of genome organization and species evolution and can also identify specific chromosomes, homologous chromosomes, chromosome rearrangements and sex chromosomes, among others. Among cichlids, FISH mapping has mostly used repeat DNA as probes, involving multigene families for rRNA [24-30] and U1 snRNA genes [31], transposons [28,32-37], and satellite DNA [36,38-40]. Besides using repeat DNA for mapping, single-copy sequences have also been mapped to the chromosomes of cichlids [31,41-43]. With the availability of completely sequenced cichlid genomes (see Cichlid Genome Consortium at http://www.bouillabase.org) and the genomes of other fish species, advances have also been made in integrating cytogenetic mapping data and genomic data [31,43].

To obtain a better understanding of the genomic organization of rRNA genes and the chromosomal evolution of cichlids, we used FISH to map the 5S rRNA genes from 41 species of representative cichlids of Etroplinae, Cichlinae and Pseudocrenilabrinae (tilapiine and haplochromine groups) and the 18S rRNA gene from 3 species of Pseudocrenilabrinae. In addition, the cytogenetic mapping data of both gene-carrying repeats (5S and 18S rDNA) were recovered from previously published data, and a genomic analysis was conducted for both gene classes from the recently available genome of the Nile tilapia, *Oreochromis niloticus*. The relationship between the variability of 5S and 18S rDNA clusters was discussed in light of the possible mechanisms that have played a role in the diversification of both rDNA clusters during cichlid fish evolution.

## Methods

### Biological samples and chromosome preparation

Animal samples were obtained from four sources (see Additional files 1 and 2): African cichlids were obtained from (i) wild stocks (mainly from Lake Malawi, East Africa) maintained at the Tropical Aquaculture Facility of the University of Maryland (TAF-UMD, USA), from (ii) Brazilian rivers (introduced tilapia species), and from (iii) aquarium shops in Botucatu, SP, Brazil; Neotropical cichlids were collected from (iv) several Brazilian and Venezuelan rivers (see Additional file 2). In total, 18 South American, 22 African and one Asian species were analyzed (see Additional files 1 and 2). The animals were collected from Brazilian rivers according to Brazilian laws for environmental protection (wild collection permit, SISBIO/15729-1). The experimental research on the animals was conducted according to the international guidelines of Sao Paulo State University (Protocol no. 34/08 - CEEA/IBB/UNESP). Mitotic chromosomes were obtained from kidneys as described by Bertollo et al. [44].

### DNA extraction, isolation of 5S and 18S rRNA gene sequences, and FISH

Genomic DNA from *O. niloticus* and Neotropical cichlids was extracted from the liver using the phenol-chloroform procedure described by Sambrook and Russel [45]. Partial sequences of 5S rDNA (including the gene plus NTS region) were obtained through polymerase chain reaction (PCR) using the primers A (5′TAC GCC CGA TCT CGT CCG ATC) and B (5′CAG GCT GGT ATG GCC GTA AGC), as described by Martins and Galetti [46]. Copies of the 18S rRNA gene from *O. niloticus* were amplified using the primers 18Sf (5′ CCG CTT TGG TGA CTC TTG AT) and 18Sr (5′CCG AGG ACC TCA CTA AAC CA), which were designed based upon the sequence of the catfish *Ictalurus punctatus* to amplify an approximately 1,400 bp DNA segment of the 18S rRNA gene [35].

PCR products were labeled by nick translation using biotin-14-dATP (Invitrogen, San Diego, CA, USA) according to the specifications of the manufacturer. For FISH on the African and Asian cichlid samples, the 5S rDNA and the 18S rDNA obtained from *O. niloticus* were use as probes. For the Neotropical representatives, 5S rDNA obtained from each species was used as the probe because heterologous probes obtained from *Astronotus ocellatus* and *Cichla kelberi* did not produce satisfactory FISH results.

The FISH procedure was performed as described by Pinkel et al. [47] with modifications based on Martins and Galetti [46] and Cabral-de-Mello et al. [31]. Images were captured with an Olympus DP71 digital camera coupled to a BX61 Olympus microscope and were optimized for brightness and contrast using Adobe Photoshop CS2.

### Analysis of 5S and 18S rRNA genes in the Oreochromis niloticus genome

The 5S rRNA (accession numbers AF478461 and AF478462) and 18S rRNA (accession number GU289229) gene sequences from *Oreochromis aureus* were used as queries in a BLASTn search against the *O. niloticus* genome (Tilapia_broad_v1 genome), annotated in the BouillaBase database (http://www.bouillabase.org) (searches conducted in Jan 2012). The putative 5S and 18S rRNA gene sequences of *O. niloticus* were analyzed using Geneious Pro 4.8.5 software [48]. The cutoff length for the hits was ≤103 nucleotides for the 5S rRNA and ≤87 nucleotides for the 18S rRNA genes; the cutoff lengths were determined from the average length of the sequences recovered. The cutoff length yielded *E* values of ≤9e-14 for 5S and ≤ 3e-17 for 18S rDNA.

The 1,000 bp upstream and 1,000 bp downstream flanking regions (FRs) of each rRNA gene copy were searched against the Repbase database [49] at the Genetic Information Research Institute (GIRI) (http://www.girinst.org/repbase/) using CENSOR software [50] to check for the presence of transposable elements (TEs).

## Results

### Cytogenetic analysis

**Figure 1:**
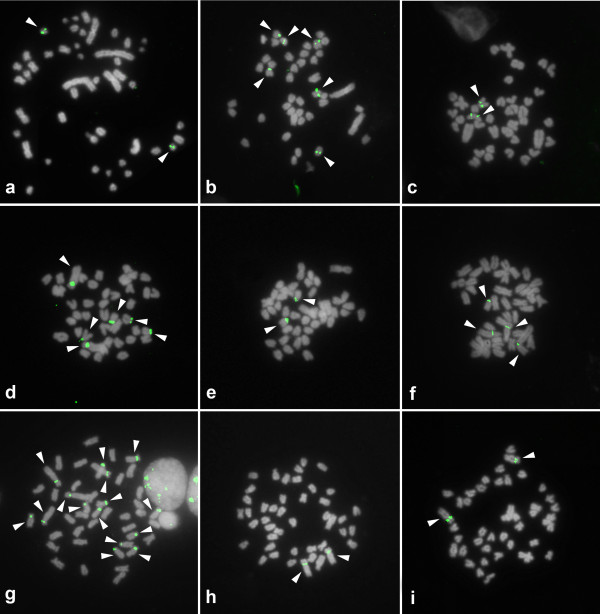
**Fluorescence*in situ*hybridization of 5S rDNA sequences to metaphasic chromosomes of Asian and African cichlids.** The 5S rDNA sequences were detected with FITC (green), the chromosomes were counterstained with DAPI, and the images were converted to grayscale. (**a**) *Etroplus maculatus*, (**b**) *Oreochromis niloticus*, (**c**) *Tilapia mariae*, (**d**) *T. mamfe*, (**e**) *Hemichromis bimaculatus*, (**f**) *Gephyrochromis moorii*, (**g**) *Astatotilapia latifasciata*, (**h**) *Melanochromis auratus*, (**i**) *Pseudotropheus tropheus*. The arrowheads indicate the 5S rDNA-bearing chromosomes. Bar = 5 μm.

**Figure 2:**
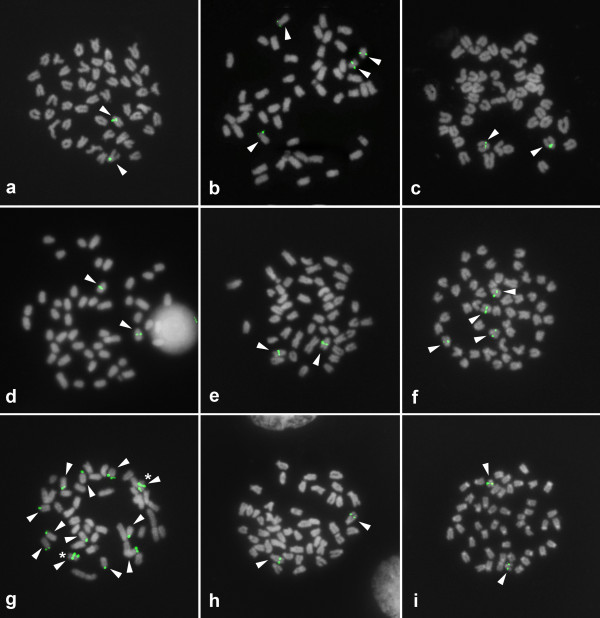
**Fluorescence*in situ*hybridization of 5S rDNA sequences to metaphasic chromosomes of Neotropical cichlids.** The 5S rDNA sequences were detected with FITC (green), the chromosomes were counterstained with DAPI, and the images were converted to grayscale. (**a**) *Retroculus lapidifer*, (**b**) *Astronotus ocellatus*, (**c**) *Cichla piquiti*, (**d**) *Geophagus proximus*, (**e**) *Satanoperca jurupari*, (**f**) *Aequidens tetramerus*, (**g**) *Laetacara dorsigera*, (**h**) *Heros efasciatus*, (**i**) *Mesonauta festivus*. The arrowheads indicate the 5S rDNA-bearing chromosomes, and the asterisks mark the chromosomes bearing two sites. Bar = 5 μm.

Basic cytogenetic data, including the diploid number and the chromosomal morphology of the analyzed species, were consistent with previously published information [24, for review]. Cytogenetic maps of 5S rDNA were obtained for 18 South American, 22 African and one Asian cichlid species, and the data are presented in the Additional files 1 and 2. Taken together with the previously published cytogenetic information for this family, the number of species investigated thus far has been updated to 23 African, 1 Asiatic and 23 Neotropical (see Additional files 1 and 2). Representative hybridized metaphasic chromosomes are shown in Figures 1 and 2. The number of sites per diploid genome ranged from 2 to 15, with the most common pattern being the presence of 2 chromosomes bearing 5S rDNA clusters. This pattern occurred in 38 species, which corresponded to ~79.0% of the samples analyzed. The highest numbers of sites were observed in the African species *Astatotilapia latifasciata* (15 sites, Figure 1g) and in the Neotropical species *Laetacara dorsigera* (14 sites, Figure 2g). These two species were excluded from the statistical analyses presented below due to their abnormally large number of 5S rDNA clusters in comparison to the other cichlid species studied.

**Figure 3:**
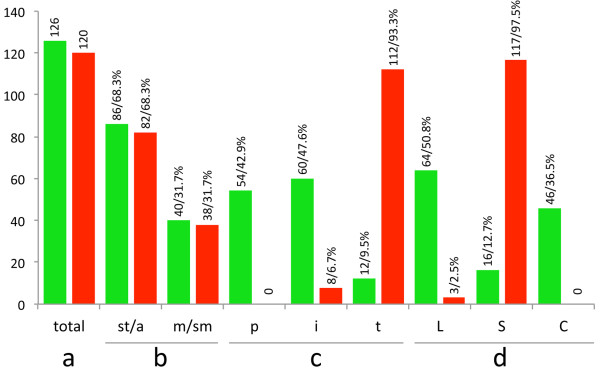
**Distribution of the number and percentage of 5S (green bars) and 18S (red bars) rDNA clusters in 48 and 41 cichlid genomes, respectively.** The species with polymorphic conditions were considered more than once. (**a**) Total number of sites, (**b**) number/percentage of sites in chromosomes with distinct morphology: telo/acrocentric (t/a), meta/submetacentric (m/sm); (**c**) number/percentage of proximal (p), interstitial (i) and terminal (t) sites; (**d**) distribution of number/percentage of sites in the distinct chromosomal arms: short (S), long (L), and in the centromeric (C) regions. The species *Astatotilapia latifasciata* and *Laetacara dorsigera* were excluded due their atypical number of 5S rDNA clusters.

The present data and the previously published results revealed an average of 2.7 sites of 5S rDNA per genome, in which ~31.7% were located in meta/submetacentric (m/sm) chromosomes and ~68.3% were in telo/acrocentric (t/a) chromosomes. With regards to the chromosomal position of these sites, ~42.9% had a proximal location (p), ~47.6% had an interstitial location (i), and ~9.5% had a terminal location (t). When examining the location of 5S rDNA on the chromosomal arms, we found that ~50.8% of sites were associated with the long arm (L) and ~12.7% were associated with the short arm (S). In addition, ~36.5% were closely mapped to the centromeric region (C), but it was impossible to distinguish the chromosomal arm on which they were positioned (see Additional files 1 and 2; Table 1; Figure 3).

**Table 1:** Characteristics of the chromosomal distributions of 5S rDNA sites in diploid genomes of cichlids*

	**African**	**Neotropical**	**Asiatic**	**Total**
Number of genomes analyzed	24**	22	01	47**
Number of sites	74	50	02	126
Average per genome	3.1	2.3	2.0	2.7
Telo/acrocentric (t/a)	42	42	02	86
Meta/submetacentric (m/sm)	32	08	00	40
Proximal (p)	50	04	00	54
Interstitial (i)	20	38	02	60
Terminal (t)	04	08	00	12
Long arm (L)	20	42	02	64
Short arm (S)	08	08	00	16
Closely associated to centromeric regions (CC)	46	00	00	46

*The species *Astatotilapia latifasciata* and *Laetacara dorsigera* were excluded due to their atypical number of 5S rDNA clusters; **Includes the polymorphic conditions detected.

Three African species were investigated for their 18S rDNA mapping (*Tilapia mamfe*, *Placidochromis electra* and *Pseudotropheus zebra*) in this study, which updates the total cichlid species analyzed to 32 (Additional files 1 and 2). The number of 18S rDNA sites varied from 2 to 6, averaging ~2.9 sites per diploid genome. The modal condition was the presence of 2 sites on one homologous chromosome pair (~63.4%). Most of the 18S rRNA gene clusters (~68.3%) were found in t/a chromosomes, and ~31.7% were located in m/sm elements. The 18S rDNA was present at the terminal location in ~93.3% of the mapped sites; only ~6.6% were interstitial, and none were present in the proximal region. Another remarkable characteristic was the association of 18S rDNA with the short arm of the chromosomes (~97.5%); only ~2.5% of the sites located on the long arm, and none associated with the centromeric regions (see Additional files 1 and 2; Table 2; Figure 3).

**Table 2:** Characteristics of the chromosomal distributions of 18S rDNA sites in diploid genomes of cichlids

	**African**	**Neotropical**	**Asiatic**	**Total**
Number of genomes analyzed	16**	24**	01	41**
Number of sites	59	59	02	120
Average per genome	3.7	2.5	2.0	2.9
Telo/acrocentric (t/a)	59	23	00	82
Meta/submetacentric (m/sm)	00	36	02	38
Proximal (p)	00	00	00	00
Interstitial (i)	00	08	00	08
Terminal (t)	59	51	02	112
Long arm (L)	00	03	00	03
Short arm (S)	59	56	02	117
Closely associated to centromeric regions (CC)	00	00	00	00

**Includes the polymorphic conditions detected.

The individual analysis of the data for the two major groups studied, the African and Neotropical cichlids, revealed particular characteristics for each group (see Additional files 1 and 2; Table 1). For the 5S rDNA, the characteristics were as follows: the average number of sites per diploid genome was 3.1 and 2.3 sites in the African and Neotropical representatives, respectively; 56.8% of sites were located in t/a chromosomes, and 43.2% of sites were located in m/sm chromosomes in the African lineage; 84% of the sites were located in t/a chromosomes, and 16% of sites in m/sm chromosomes in the Neotropical cichlids; the preferential location of the sites was proximal (67.6%) in Africans and interstitial in Neotropical cichlids (76.0%); in the Africans, most of the sites were closely mapped to the centromeric region (71.9%), while for Neotropicals, the 5S rDNA was mostly mapped to the long chromosomal arms (84.0%). For the 18S rDNA, the characteristics were as follows: the average number of sites per diploid genome was 3.7 and 2.5 sites in the African and Neotropical representatives, respectively; all of the sites observed for the 18S rDNA in the African cichlids were located in the terminal regions of the short arms of t/a chromosomes (see Additional file 1; Table 2); in the Neotropical species, a more variable pattern for the position of this marker was observed with locations in both the m/sm and t/a chromosomes; the precise location was primarily in the terminal region of the short arm (see Additional file 2; Table 2).

**Figure 4:**
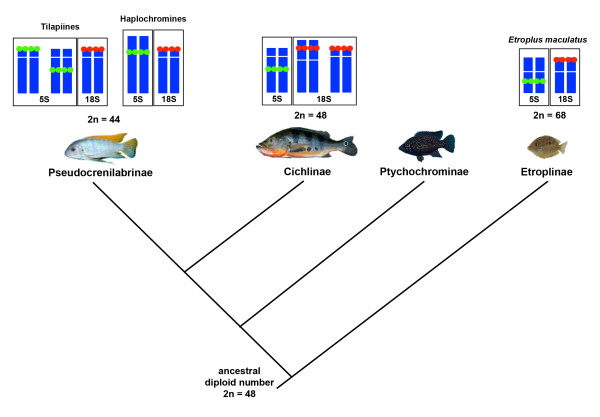
**Synthetic view of the most frequent chromosomal locations of rDNA plotted on the cladogram of the cichlid family.** The tree was based on the phylogeny proposed by Sparks and Smith (2004).

Besides the general differences between the two cichlid lineages, in African species, variations in the distribution of 5S rDNA were observed among tilapiines and haplochromines. In tilapiines (Figure 1b-d), the 5S rDNA sites were generally located on small t/a chromosomes. In haplochromines (Figure 1f-i), these gene copies were located on the largest m/sm chromosome pair in all of the species analyzed (see Additional file 1; Table 2) with additional sites observed in *Astatotilapia latifasciata* (Figure 1g) and *Gephyrochromis moorii* (Figure 1f). A synthetic view of the possible modal number and chromosome locations for 5S and 18S rDNA clusters in the distinct lineages of cichlids is shown in Figure 4.

### Analysis of 5S and 18S rRNA genes in the O. niloticus genome

**Figure 5:**
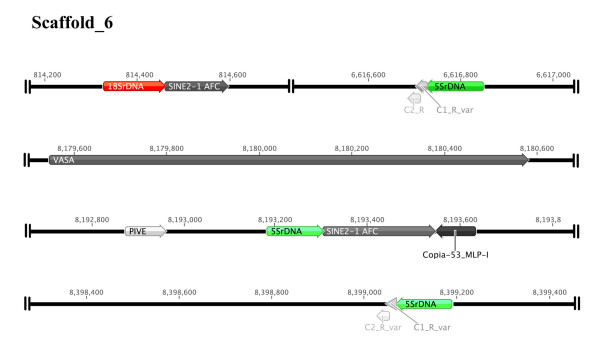
**The organization of rRNA genes and TEs on scaffold 6 of the*Oreochromis niloticus*genome.** The 5S and 18S rRNA genes, putative TEs (SINE2-1_AFC, PIVE, Copia-53_MLp-1 and SINE_FR2), and the vasa gene are indicated by arrows. C1 and C2 represent conserved repeats in the NTS of the 5S rRNA type I gene.

The results for the 5S rRNA gene BLASTn search of the BouillaBase database retrieved 59 copies that were distributed on 42 scaffolds, and the search for the18S rRNA gene retrieved 38 copies that were distributed on 31 scaffolds (see Additional file 3). For each result, the putative copy was ranked according to its sequence length and E-value. Scaffold 6 was the only one to harbor putative 5S and 18S rRNA gene copies (Figure 5). Most of the 18S rRNA sequences recovered were partial copies of the gene (see Additional file 4). Although previous studies detected two types of 5S rDNA repeats (type I and type II) in the *O. niloticus* genome [25], only type I copies were recovered in the present analysis (see Additional file 5). Moreover, the analysis of the FRs of the rRNA genes (see Additional files 6 and 7) detected the presence of transposable elements (TEs) that flanked a high number of rRNA gene copies, with a predominance of SINEs in the FRs of 5S rRNA gene sequences (Figure 5).

## Discussion

### Comparative cytogenetic mapping of rRNA genes among cichlids

Although variations in the number of rRNA gene clusters were frequent among the cichlid groups analyzed, the presence of two clusters in homologous chromosomes was the most common condition observed in 79.0% and 63.4% of the 5S and 18S rRNA genes, respectively. Furthermore, variations in the specific chromosome positions of the rRNA genes were observed in all of the cichlid groups analyzed.

The presence of 5S rDNA clusters in the interstitial or proximal position was frequently observed here for tilapiines, haplochromines and cichlines, as has previously been observed in several other fish groups (for review, [51]). These data support the hypothesis that the presence of 5S rDNA sites that are non-terminally located could represent the ancestral condition of the chromosomal organization of 5S rRNA genes, especially in light of the distinct phylogenetic lineages and sister groups that were studied. Among other vertebrates, 5S rDNA in the interstitial position is also a typical pattern, as seen in mammals and amphibians [52-54]. In invertebrates, the location of 5S rDNA has been well studied. For example, in some Acrididae grasshoppers, most of the sites (96.6%) are located in either the proximal or the interstitial position [5]. In the African cichlid species, some clusters of 5S rDNA were observed in the proximal position closely associated with the centromere. This phenomenon was seen most commonly in the haplochromine cichlid samples, suggesting that it could be related to a specific chromosomal rearrangement in the largest m/sm chromosomal pair that occurred before the diversification of this group. In fact, it was reported that fusion processes generated this chromosome in the African cichlids, and at least one fusion occurred in haplochromines [36] (Figure 4). In contrast, the 18S rDNA clusters were mostly located at the terminal position in cichlids and in fish as a whole. Exceptions were observed in only four Cichlinae representatives belonging to distinct tribes and seem to be related to the occurrence of specific chromosomal rearrangements. Although two-color FISH for 18S and 5S rDNA was not performed, it is clear that in general, these two gene clusters are located on distinct chromosomes, as has been commonly observed in other fishes [55].

The diversity of Cichlinae species is lower than that of Pseudocrenilabrinae species, but the cytogenetic analyses have shown higher levels of chromosome variations in cichlines than in Pseudocrenilabrinae species (for review, [24,25]). However, the comparative analysis of rDNA cluster numbers between Cichlinae and Pseudocrenilabrinae clades revealed higher levels of variation in the African cichlids, considering the number of clusters per genome. Thus, the genomic dynamics for the spreading of clusters seems to be greater in the African group. Although Cichlinae has more dynamic karyotypes possibly due to the occurrence of macro-chromosomal rearrangements, the Pseudocrenilabrinae species exhibit greater dynamism at microgenomic levels, at least with regards to rRNA genes.

The average cluster number per genome is slightly higher for 18S rDNA, and the more intense dispersion of these genes seems to be related to their common presence in the terminal regions of the chromosomes. The amplification/dispersion of rDNA segments could be mediated by extrachromosomal circular DNA (eccDNA), transposable elements and/or heterologous recombination [56,57], which cause the origin of new loci either followed or not followed by the deletion of the original sites. Additionally, the genomic dynamism of terminal regions of the chromosomes could favor transposition events, leading to the dispersion of segments. In contrast, the interstitial and proximal chromatid environment occupied by the 5S rRNA genes seems to be a more stable chromosomal region than the terminal position, thereby avoiding major genomic changes that could generate the dispersion of these copies.

Despite the intense and particular genomic dynamics of rDNA repeats (see the next topic of discussion) that masks most of the phylogenetic relationships of taxa, some modal patterns based on the 5S rDNA chromosomal clusters of cichlids can be observed. This could indicate a common origin, with the most remarkable example being the clusters closely associated with the centromere of pair 1 in haplochromines. Additionally, the presence of one interstitial cluster in a t/a chromosome pair occurs in all Cichlinae members; this chromosome is also detected in some African species (Pseudocrenilabrinae) and in the Asian *E. maculatus* (Etroplinae) (Figure 4). However, the Perciformes Cichlidae sister families also include the presence of one interstitial cluster in a t/a but have other diverse patterns of 5S rDNA chromosomal cluster distributions, as observed in Haemulidae [58,59] and Pomacentridae [60], making it difficult to identify plesiomorphic patterns.

### Genomic organization of rRNA genes with a focus on Oreochromis niloticus

The extensive variation in the number and chromosomal position of rDNA clusters that was observed among the cichlids analyzed seems to be related to the intense evolutionary dynamics of repeated units of rRNA genes that generates divergent patterns of chromosomal distribution even among closely related species. Based on data from several organisms, including fish, rRNA gene families seem to evolve according to a combination of the evolutionary processes of birth-and-death and concerted evolution [6,61], which could explain the variability observed among related taxa.

The association of rRNA genes and transposable elements observed here in the *O. niloticus* genome has been extensively reported among animals and plants [56,62-66] and could be responsible for the chromosomal cluster variation observed. Drouin [67] shows that some of the expressed 5S rRNA genes observed in the mouse and rat genomes were derived from the retrotransposition of 5S rRNA transcripts. It seems plausible that the activity of TEs is one possible source for rRNA gene movement that could generate the spreading of rDNA clusters as was observed in the cytogenetic analysis. This is interesting because the cytogenetic mapping of rRNA genes has been frequently discussed from a phylogenetic perspective. Such assumptions should be carefully addressed because the clustering of rRNA genes seems to reflect their intense and particular evolutionary pathway and not the evolutionary history of taxa.

Contrary to the cytogenetic mapping of the rRNA genes that yielded major chromosomal clusters for these genes, the analysis of the *O. niloticus* genome database demonstrated only spread copies of both 5S and 18S rRNA. The data obtained suggest that genome sequencing data are not informative for clarifying the exact copy number and the correct genomic organization of repeat DNA. These results are the consequence of the exclusion of many repeated sequences prior to genomic assembly in an attempt to facilitate the assembly procedure. However, the information collected provides a perspective on rRNA gene spreading in cichlids adding new information about the organization of rDNAs, at least in *O. niloticus*, not based only in chromosomal analysis.

## Conclusions

The chromosomal distribution of rRNA genes seems to reflect an intense and particular evolutionary pathway that does not follow the evolutionary history of taxa in most cases and seems to be influenced by the presence of TEs in the FRs of the rRNA genes. Additionally, the distinct patterns of organization/spreading of the two multigene families indicate distinct evolutionary forces acting in the diversification of these elements in cichlid genomes, which could reflect the association of distinct TEs. The organization of rRNA and other repeated genes has been hindered in completely sequenced genomes due to the difficulty of assembling them into a genome. Thus, the integration of cytogenetic mapping and genomic sequencing data, as reported here, leads to a better understanding of the genomic organization and evolution of repeated sequences.

## Abbreviations

FISH: Fluorescence *in situ* hybridization; FR: Flanking region; ITS: Internal transcribed spacers; m/sm: Meta/submetacentric; NTS: Non-transcribed spacer; rRNA: Ribosomal RNA; t/a: Telo/acrocentric; TE: Transposable element.

## Competing interests

The authors declare that they have no competing interests.

## Authors’ contributions

RTN conducted the chromosome preparations, the molecular cytogenetics experiments with rRNA gene probes, the *in silico* analyses, the interpretation of the data, and drafting of the manuscript. GTV conceived the initial *in silico* analysis, interpreted the data and revised the manuscript. DCCM assisted in the chromosome preparations, molecular cytogenetics, interpretation of the data and revision of the manuscript. PCV conducted the chromosome preparations from the South American species and helped in the preparation of the manuscript. CM conceived the study, participated in its design and coordination and helped to draft the manuscript. All authors read and approved the final manuscript.

## Supplementary Material

Additional file 1 **Title and description of data: Investigated Asian and African cichlids**[68,69]. Click here for file

Additional file 2 Title and description of data: Investigated Neotropical cichlids. Click here for file

Additional file 3 **Title and description of data: Number of 5S and 18S rRNA gene copies retrieved from the*Oreochromis niloticus*genome at the BouillaBase database** (http://www.bouillabase.org). Click here for file

Additional file 4 **Title and description of data: Alignment of the 18S rRNA gene copies retrieved from the*Oreochromis niloticus*genome at the BouillaBase database (**http://www.bouillabase.org**).** The nucleotides are identified by different colors, and the black blocks under the alignment indicate the consensus sequence. Click here for file

Additional file 5 **Title and description of data: Alignment of the 5S rRNA gene copies retrieved from the*Oreochromis niloticus*genome at the BouillaBase database (**http://www.bouillabase.org**).** The nucleotides are identified by different colors, and the black blocks under the alignment indicate the consensus sequence. Click here for file

Additional file 6 **Title and description of data: Identified transposable elements (TEs) in the flanking regions (FRs) of 5S rRNA gene copies retrieved from the*O. niloticus*genome and their similarity (%) level to sequences deposited in Repbase.** d1, d2…, u1, u2… indicate the different copies detected. The position of the TE is indicated by “d” - downstream of the gene or “u” - upstream of the gene. R indicates reverse copies of the TE. Click here for file

Additional file 7 **Title and description of data: Identified transposable elements (TEs) in the flanking regions (FRs) of 18S rRNA gene copies retrieved from the*O. niloticus*genome and their similarity (%) level to sequences deposited in Repbase.** The position of the TE is indicated by “d” - downstream of the gene or “u” - upstream of the gene. d1, d2…, u1, u2… indicate the different copies detected. R indicates reverse copies of the TE. Click here for file
