# Supplementary material for: Evolutionary dynamics of rRNA gene clusters in cichlid fish
Source: BMC Evol Biol. 2012 Oct 5;12:198. doi: 10.1186/1471-2148-12-198 (PMC3503869; doi:10.1186/1471-2148-12-198)
Supplement: Additional file 1 — Title and description of data: Investigated Asian and African cichlids[68,69]. [file 1471-2148-12-198-S1.pdf]

Additional file 1. Investigated Asian and African cichlids.

Major groups and species	Origin of samples	2n	5S rDNA number and position	Chromosomal arm	Reference	45S rDNA number and position	Chromosomal arm	References
**Etroplinae**								
Etroplus maculatus	Petshop, Botucatu, SP, Brazil	46	2t/a(i)	L	This work	2m/sm(t)	S	[24]
**Pseudocrenilabrinae**								
Tilapiines								
Oreochromis aureus	TAF-UMD, USA	44	2t/a(p)	CC	This work	2t/a(t)		
*Oreochromis mossambicus**	TAF-UMD, USA; Gaoyao Aquac. Germplasm Conserv. Station	44	2t/a(i); 4t/a(i)+2t/a(t)	L; 4L+2S	This work, [68]	3t/a(t); 6t/a(t)	S; S	[68, 24]
*Oreochromis niloticus**	Tietê river, Botucatu, SP, Brazil; Aquac. Facility, Dalhousie Univ., Canada	44	4t/a(i)+2t/a(p); 4t/a(i)+4t/a(p)	4L+2S; 4L+2S+2CC	This work, [25]	6t/a(t)	S	[24, 26]
Oreochromis tanganica	TAF-UMD, USA	44	2t/a(p)	CC	This work			
Oreochromis urolepis hornorum	Gaoyao Aquac. Germplasm Conserv. Station	44	4t/a(i)+2t/a(t)	4L+2S	[68]	6t/a(t)	S	[25]
Tilapia mariae	TAF-UMD, USA	40	2t/a(i)	L	This work	2t/a(t)	S	[24]
Tilapia mamfe	TAF-UMD, USA	44	2m/sm(p)+4t/a(p)	CC	This work	2t/a(t)	S	This work
Hemichromines								
Hemichromis bimaculatus	Petshop, Botucatu, SP, Brazil	44	2t/a(p)	CC	This work	2t/a(t)	S	[24]
Haplochromines								
Astatotilapia burtoni	TAF-UMD, USA	40	2m/sm(p)	CC	This work	2t/a(t)	S	[24]
Gephyrochromis moorii	Petshop, Botucatu, SP, Brazil	44	2m/sm(p)+2t/a(p)	CC	This work			
Haplochromis venustus	TAF-UMD, USA	44	2m/sm(p)	CC	This work			
Haplochromis livingstonii	TAF-UMD, USA	44	2m/sm(p)	CC	This work			
Astatotilapia latifasciata[#]	Petshop, Botucatu, SP, Brazil	44	2m/sm(p)+13t/a(p)	CC	This work	4t/a(t); 6t/a(t)	S; S	[24, 69]
Labeotropheus trewavasae	TAF-UMD, USA	44	2m/sm(p)	CC	This work	2t/a(t)	S	[24]
Melanochromis auratus	TAF-UMD, USA	44	2m/sm(p)	CC	This work	2t/a(t)	S	[24]
Placidochromis electra	TAF-UMD, USA	44	2m/sm(p)	CC	This work	6t/a(t)	S	This work
Metriaclima gold zebra	TAF-UMD, USA	44	2m/sm(p)	CC	This work	2t/a(t)	S	[24]
Metriaclima lombardoi	TAF-UMD, USA	44	2m/sm(p)	CC	This work	2t/a(t)	S	[24]
Metriaclima callainos	TAF-UMD, USA	44	2m/sm(p)	CC	This work			

Metriaclima benetos	TAF-UMD, USA	44	2m/sm(p)	CC	This work			
Pseudotropheus sp	TAF-UMD, USA	44	2m/sm(p)	CC	This work			
Pseudotropheus tropheus	TAF-UMD, USA	44	2m/sm(p)	CC	This work			
Pseudotropheus zebra	TAF-UMD, USA	44	2m/sm(p)	CC	This work	4t/a(t)	S	This work

2n, diploid number; t/a, telo/acrocentric; m/sm, meta/submetacentric; L, long arm; S, short arm; CC, closely associated to centromeric region; (p), proximal; (i), interstitial; (t), terminal. The (*) indicates species with polymorphism for 5S rDNA sites, and (#) species with polymorphism for 45S rDNA. The polymorphic conditions are separated by (;).
