# Supplementary material for: Evolutionary dynamics of rRNA gene clusters in cichlid fish
Source: BMC Evol Biol. 2012 Oct 5;12:198. doi: 10.1186/1471-2148-12-198 (PMC3503869; doi:10.1186/1471-2148-12-198)
Supplement: Additional file 2 — Title and description of data: Investigated Neotropical cichlids. [file 1471-2148-12-198-S2.pdf]

Additional file 2. Investigated Neotropical cichlids.

Major groups and species	Origin of samples	2n	5S rDNA number and position	Chromosomal arm	Reference	45S rDNA number and position	Chromosomal arm	References
**Cichlinae**								
**Retroculini**								
Retroculus lapidifer	Araguaia river, Barra do.Garças, MT, Brazil	48	2t/a(p)	L	This work	2t/a(t)	S	[24]
**Astronotini**								
Astronotus ocellatus	Araguaia river, Barra do Garças, MT, Brazil	48	2m/sm(t)+2t/a(i)	2S+2L	This work	2m/sm(i)	S	[24]
**Cichlini**								
Cichla piquiti	Araguaia river, Barra do Garças, MT, Brazil	48	2t/a(p)	L	This work			
Cichla kelberi	Araguaia river, Barra do Garças, MT, Brazil	48	2t/a(i)	L	This work, [35]	2t/a(t)	L	[35, 24]
**Chaetobranchini**								
Chaetobranchus flavescens	Araguaia river, Barra do Garças, MT, Brazil	48	2t/a(i)	L	This work	2m/sm(i)	S	[24]
**Geophagini**								
Geophagus brasiliensis	Araguaia river, Barra do Garças, MT, Brazil	48	2t/a(i)	L	This work, [27]	2t/a(t)	S	[27]
Geophagus proximus	Araguaia river, Barra do Garças, MT, Brazil	48	2t/a(i)	L	This work			
Geophagus surinamensis	Orinoco river, Caicara, Venezuela	48	2t/a(i)	L	This work			
Bitodoma cupido	Araguaia river, Barra do Garças, MT, Brazil	48	2t/a(i)	L	This work	2m/sm(t)	S	[24]
Crenicichla lepidota	São Gonçalo stream and Polegar lake, RS, Brazil	48	4t/a(i)	L	[30]	2m/sm(i)	S	[30]
Satanoperca jurupari	Araguaia river, Barra do Garças, MT, Brazil	48	2t/a(i)	L	This work	2t/a(t)	S	[24]
**Cichlasomatini**								
Aequidens tetramerus	Araguaia river, Barra do Garças, MT, Brazil	48	4t/a(i)	L	This work	2t/a(t)	S	[24]
Aequidens plagiozonatus	Araguaia river, Barra do Garças, MT, Brazil	48	2t/a(i)	L	This work			

Cichlasoma facetum	Tarumã river, Ponta Grossa, PR, Brasil	48	2t/a(i)	L	[27]	2t/a(t)	S	[27]
Cichlasoma nigrofasciatum	Araguaia river, Barra do Garças, MT, Brazil	48	2t/a(i)	L	This work			
Cichlasoma paranaense	Carrapato stream, Penápolis, SP, Brazil	48	2t/a(i)	L	This work			
Laetacara dorsigera	Araguaia river, Barra do Garças, MT, Brazil	44	2t/a(p)+2t/a(i)+10t/a (p)	2L+12CC	This work	2t/a(t)	S	[24]
**Heroini**								
Heros efasciatus	Araguaia river, Barra do Garças, MT, Brazil	48	2t/a(i)	L	This work	2t/a(t)	S	[24]
Mesonauta festivus	Araguaia river, Barra do Garças, MT, Brazil	48	2t/a(i)	L	This work	6t/a(t)	S	[24]
Pterophyllum scalare	Araguaia river, Barra do Garças, MT, Brazil	48	2t/a(i)	L	This work	2m/sm(t)	S	[24]
Symphysodon aequifasciatus#	Tefé river, AM, Brazil	60	2m/sm(t)	S	[28]	2m/sm(t); 3m/sm(t)	S	[28]
Symphysodon discus#	Negro river, AM, Brazil	60	2m/sm(t)	S	[28]	2m/sm(t); 3m/sm(t); 4m/sm(t); 5m/sm(t)	S	[28]
Symphysodon haraldi#	Manacapuru river, AM, Brazil	60	2m/sm(t)	S	[28]	2m/sm(t); 2m/sm(t)+1t/a(i); 2m/sm(t)+1m/sm(i)	S; 2S+1L; S	[28]

2n, diploid number; t/a, telo/acrocentric; m/sm, meta/submetacentric; L, long arm; S, short arm; CC, closely associated to centromeric region; (p), proximal; (i), interstitial; (t), terminal. The (#) indicates species with polymorphism for 45S rDNA. The polymorphic conditions are separated by (;).
