# Supplementary material for: Evolutionary dynamics of rRNA gene clusters in cichlid fish
Source: BMC Evol Biol. 2012 Oct 5;12:198. doi: 10.1186/1471-2148-12-198 (PMC3503869; doi:10.1186/1471-2148-12-198)
Supplement: Additional file 3 — Title and description of data: Number of 5S and 18S rRNA gene copies retrieved from theOreochromis niloticusgenome at the BouillaBase database (http://www.bouillabase.org). [file 1471-2148-12-198-S3.pdf]

Additional file 3: Number of 5S and 18S rRNA gene copies retrieved from the *Oreochromis niloticus* genome database at the BouillaBase (http://cichlid.umd.edu/cichlidlabs/kocherlab/bouillabase.html).

Scaffold	Number of 5S rRNA gene copies
Scaffold_0	1
Scaffold_3	1
Scaffold_4	1
Scaffold_6	3
Scaffold_7	2
Scaffold_9	1
Scaffold_14	1
Scaffold_22	1
Scaffold_26	1
Scaffold_54	1
Scaffold_55	2
Scaffold_66	1
Scaffold_69	1
Scaffold_83	1
Scaffold_90	1
Scaffold_101	1
Scaffold_116	1
Scaffold_117	1
Scaffold_148	1
Scaffold_164	4
Scaffold_191	9
Scaffold_219	1
Scaffold_237	1
Scaffold_253	1
Scaffold_261	1
Scaffold_263	1
Scaffold_286	1
Scaffold_328	1
Scaffold_397	1
Scaffold_408	1
Scaffold_512	1
Scaffold_536	1
Scaffold_624	1
Scaffold_677	2
Scaffold_688	1
Scaffold_768	3
Scaffold_791	1
Scaffold_813	2
Scaffold_1031	1
Scaffold_1299	1
Scaffold_1430	1
Scaffold_1626	1
**total**	**59**

Scaffold	Number of 18S rRNA gene copies
Scaffold_1	2
Scaffold_6	1
Scaffold_8	1
Scaffold_13	4
Scaffold_19	3
Scaffold_38	1
Scaffold_40	1
Scaffold_44	1
Scaffold_58	1
Scaffold_61	1
Scaffold_86	1
Scaffold_104	1
Scaffold_114	1
Scaffold_118	1
Scaffold_136	1
Scaffold_143	1
Scaffold_169	1
Scaffold_173	1
Scaffold_181	1
Scaffold_182	1
Scaffold_185	1
Scaffold_197	1
Scaffold_201	1
Scaffold_214	1
Scaffold_245	1
Scaffold_250	1
Scaffold_515	1
Scaffold_516	2
Scaffold_834	1
Scaffold_1005	1
Scaffold_1544	1
**total**	**38**
