# Supplementary material for: Evolutionary dynamics of rRNA gene clusters in cichlid fish
Source: BMC Evol Biol. 2012 Oct 5;12:198. doi: 10.1186/1471-2148-12-198 (PMC3503869; doi:10.1186/1471-2148-12-198)

Additional file 4: Alignment of the 18S rRNA gene copies retrieved from the *Oreochromis niloticus* genome at the BouillaBase database (http://cichlid.umd.edu/cichlidlabs/kocherlab/bouillabase.html). The nucleotides are identified by different colors, and the black blocks under the alignment indicate the consensus sequence.

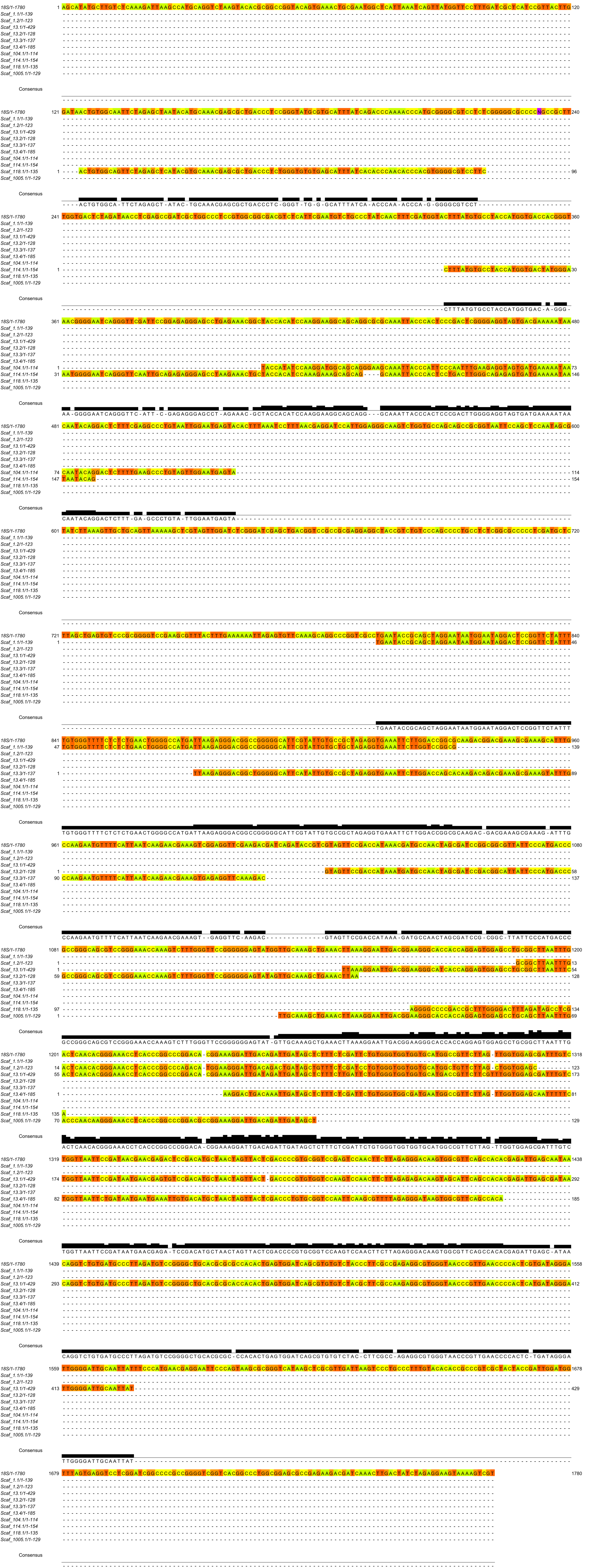

Supplement: Additional file 4 — Title and description of data: Alignment of the 18S rRNA gene copies retrieved from theOreochromis niloticusgenome at the BouillaBase database (http://www.bouillabase.org). The nucleotides are identified by different colors, and the black blocks under the alignment indicate the consensus sequence. [file 1471-2148-12-198-S4.pdf]
