# Supplementary material for: Evolutionary dynamics of rRNA gene clusters in cichlid fish
Source: BMC Evol Biol. 2012 Oct 5;12:198. doi: 10.1186/1471-2148-12-198 (PMC3503869; doi:10.1186/1471-2148-12-198)
Supplement: Additional file 6 — Title and description of data: Identified transposable elements (TEs) in the flanking regions (FRs) of 5S rRNA gene copies retrieved from theO. niloticusgenome and their similarity (%) level to sequences deposited in Repbase. d1, d2…, u1, u2… indicate the different copies detected. The position of the TE is indicated by “d” - downstream of the gene or “u” - upstream of the gene. R indicates reverse copies of the TE. [file 1471-2148-12-198-S6.pdf]

Additional file 6: Identified transposable elements (TEs) in the flanking regions (FRs) of 5S rRNA gene copies retrieved from the *O. niloticus* genome and their similarity (%) level to sequences deposited in Repbase. d1, d2…, u1, u2… indicate the different copies detected. The position of the TE is indicated by "d" - downstream of the gene or "u" - upstream of the gene. R indicates reverse copies of the TE.

Scaffolds	FRs	TEs	Similarity (%)
Scaf_0	d (R)	SINE2-1_AFC	0.7982
	u (R)	-	-
Scaf_4	d	L2-41 DRe	0.7022
	u	SINE2-1 AFC	0.8513
		MuDr-2x_AP	0.8525
Scaf_6	d.1 (R)	-	-
	u.1 (R)	LINE2CI_CE	0.8367
	d.2	PIVE	0.7595
	u.2	SINE2-1 AFC	0.8655
		Copia-53 MLP-I	0.7711
	d.3	-	-
	u.3	-	-
Scaf_7	d.1	MUDRAVI2	0.7500
		Kolobok-15_HM	0.8333
	u.1	-	-
	d.2 (R)	DNA_TTAA0-7-	0.7014
		DR	0.7015
		DNA -8-24 DR	0.7222
		DNA -8-24 DR	
	u.2 (R)	hAT-N21_DR	0.7969
Scaf_9	d	-	-
	u	CR1-29_DR	0.8627
		RNERVK23	0.7288
Scaf_14	d	MAUI	0.7411
		Tc1-1_ACar	0.9355
		Mariner-2_DYa	0.7054
	u	SINE2-1 AFC	0.8626
Scaf_22	d	AeTango2	0.7547
	u	SINE2-1 AFC	0.8313
		SINE2-1 AFC	0.8679
		MAUI	0.7317
Scaf_26	d	DNA-4-5 DR	0.8222
		CR1-1 DAn	0.7363
	u	SINE2-1 AFC	0.8503
		Gypsy-22_SB-I	0.7719
Scaf_54	d (R)	RTE-1 TD	0.7573
		RTE-2 AFC	0.7967

		RTE-2 AFC	0.7861
		TC1 DR3	0.8457
		TC1 DR3	0.8077
		SMAR 12	0.9268
	u (R)	SINE2-1 AFC	0.8699
		SMAR 12	0.8307
Scaf_55	d (R)	SINE2-1 AFC	0.8642
	u (R)	-	-
Scaf_66	d (R)	SINE2-1 AFC	0.8519
	u (R)	MuDR-PGr	0.7170
		Rex5	0.8136
		RSINE2	0.9355
Scaf_69	d	CR1-25_HM	0.7778
		TE-X-4_DR	0.7174
		5S_CPo	0.9796
	u	TC1 DR3	0.8438
		TC1DR3	0.8039
Scaf_83	d	-	-
	u	SINE_FR2	0.8045
Scaf_90	d	Tc1-1 PM	0.7057
	u	SINE2-1 AFC	0.8955
		Tc1-3 Xt	0.7000
		hAT-1 NV	0.7041
Scaf_101	u	MYSERV16_I	0.7778
	d	REX1-7_XT	0.8000
		CER15-1-I_CE	0.8333
Scaf_116	d(R)	REP522	0.7321
		SINE2-1 AFC	0.8230
	u(R)	SINE2-1 AFC	0.8134
		EnSpm2 TM	0.7474
Scaf_117	d	Gypsy-39	0.8043
	u	Polinton-2_HM	0.7442
Scaf_148	d	Tc1-7 DR	0.7273
		Tc1-FR3	0.8116
	u	SINE2-1 AFC	0.8346
		Tc1-9 Xt	0.7411
Scaf_164	d.1 (R)	Copia 18-PTR_I	0.9310
		EnSpm-3-HV	0.7759
		SINE2-1 AFC	0.8185
	u.1 (R)	hATm-1_CS	0.8571
	d.2(R)	EnSpm-13-SBi	0.8537
		Ginger2-1 NV	0.7612
		Helitron-2 DR	0.7099
	u.2(R)	5S_CPo	0.7900
		Meg_PVa	0.8393
	d.3 (R)	5S_CPo	0.7900

		Meg_PVa	0.8393
		Polinton-2_CB	0.7600
	u.3 (R)	5S_CPo	0.9576
		Meg_PVa	0.7463
	d.4	Meg_PVa	0.8393
		Polinton-2_CB	0.7600
		5S_CPo	0.9661
	u.4	Meg_PVa	0.7463
		DNA-3-5_DR	0.7679
Scaf_191	d.1	Daphne-18_HM	0.7500
	u.1	L1-23_ACar	0.7869
	d.2	hAT-1_SM	0.8077
	u.2	-	-
	d.4 (R)	-	-
	u.4 (R)	HARB-N1_STu	0.9062
	d.5	DNA-8-5_DR	0.8600
	u.5	Tc1-10 Xt	0.9704
	d.6	-	-
	u.6	hAT-N74_DR	0.7952
		CHAPLIN5_FR	0.7800
	d.7	MuDR-11n_VV	0.8269
	u.7	Sola2-3_DPu	0.7526
		CR1-8_CQ	0.7869
		MuDR-7_Mad	0.7761
		hAT-N23_DR	0.7281
Scaf_219	d	-	-
	u	SINE2-1 AFC	0.8375
Scaf_237	d	hAT-N74_DR	0.8000
		SMAR 12	0.7692
	u	hATx-3 SM	0.7935
		LTR16-Pca	0.7895
Scaf_253	d	-	-
	u	SINE2-1 AFC	0.8534
		hAT-N26-DR	0.7872
Scaf_261	d	MEG-RS	0.8333
	u	-	-
Scaf_263	d	Gypsy-33_GA-I	0.7826
		Gypsy-33_GA-I	0.7395
		MAUI	0.7500
	u	Expander	0.7638
		Expander	0.7819
Scaf_286	d	DNA-6-N7_DR	0.7039
		DNA-6-N7_DR	0.7526
		COP20_I_MT	0.8246
	u	SINE2-1 AFC	0.8413
		EnSpm2-TM	0.7426

Scaf_328	d	HERV17	0.7290
	u	SINE2-1 AFC	0.8710
		MAUI	0.7188
Scaf_397	d	TC1_FR3	0.7206
	u	SINE2-1 AFC	0.8452
		Gypsy-256_AA-I	0.7660
Scaf_408	d	SINE2-1_AFC	0.8375
	u	SINE_TE	0.8804
		SINE_AFC	0.8991
Scaf_512	d	Helitron-2_HM	0.7887
		MuDR-6_STu	0.7750
	u	TAG2	0.7674
Scaf_536	d (R)	CRI-3 FR	0.7211
		DNA8-9_CQ	0.8197
	u (R)	Gypsy-24-LTR_SP	0.8837
Scaf_624	d (R)	Mariner-8_Dan	0.8226
		Rex1-7_XT	0.7350
		SINE2-1_AFC	0.8492
	u (R)	REX1-2_XT	0.7200
		piggyBack-N2_XT	0.8372
		Gypsy-8_Dan-I	0.9062
Scaf_677	d.2	Tc1-11Xt	0.7857
	u.2	-	-
Scaf_688	d (R)	L2-1_DR	0.8919
		MAUI	0.7849
	u (R)	Tc1-10ry	0.7297
		Tc1-1_PM	0.7593
		Penelope-1_AFC	0.9084
Scaf_768	d.3 (R)	SINE_AFC	0.9127
		LTR125_MD	0.8367
	u.3 (R)	TE-X-4_DR	0.8889
Scaf_791	d	ENSPM-6_DR	0.8857
		CHAPLIN-4_FR	0.7467
	u	CR1-29_DR	0.8393
		LTR112b_MD	0.8611
		L1-13_DR	0.7351
		RTE-2_AFC	0.8272
		DNA-8-14_DR	0.8000
Scaf_813	d.1	Gypsy-14_SMo-I	0.7971
		Gypsy-134_ZM-I	0.7627
	u.1	-	-
Scaf_1430	d (R)	SINE_AFC	0.8810
	u (R)	-	-
Scaf_1626	d	CHAPLIN4_FR	0.7333
	u	CR1-31_DR	0.8939
