# Supplementary material for: Evolutionary dynamics of rRNA gene clusters in cichlid fish
Source: BMC Evol Biol. 2012 Oct 5;12:198. doi: 10.1186/1471-2148-12-198 (PMC3503869; doi:10.1186/1471-2148-12-198)
Supplement: Additional file 7 — Title and description of data: Identified transposable elements (TEs) in the flanking regions (FRs) of 18S rRNA gene copies retrieved from theO. niloticusgenome and their similarity (%) level to sequences deposited in Repbase. The position of the TE is indicated by “d” - downstream of the gene or “u” - upstream of the gene. d1, d2…, u1, u2… indicate the different copies detected. R indicates reverse copies of the TE. [file 1471-2148-12-198-S7.pdf]

Additional file 7: Identified transposable elements (TEs) in the flanking regions (FRs) of 18S rRNA gene copies retrieved from the *O. niloticus* genome and their similarity (%) level to sequences deposited in Repbase. The position of the TE is indicated by "d" - downstream of the gene or "u" - upstream of the gene. d1, d2…, u1, u2… indicate the different copies detected. R indicates reverse copies of the TE.

Scaffolds	FR	TEs	Similarity (%)
Scaf_1	d.1	Kiri-13_AAe	0.7333
		hAT-N9_SBi	0.7603
	u.1	Dong_FR2	0.8288
		ATCOPIA8BI	0.8200
		Tc1-9_Xt	0.7794
	d.2	SINE2-1 AFC	0.8455
	u.2	Copia-75_SB-I	0.8103
		Transib-3_HM	0.7963
Scaf_6	d	-	-
	u	SINE2-1 AFC	0.8819
Scaf_8	d	Gypsy-26-I_DR	0.7179
		Gypsy-137_GM-I	0.7917
		Gypsy-172_AA-I	0.8611
	u	-	-
Scaf_13	d.1 (R)	Copia25-PTR_I	0.7258
	u.1 (R)	piggyback-N3_DR	0.8182
	d.2 (R)	piggyback-N3_DR	0.8222
	u.2 (R)	-	-
	d.3 (R)	piggyback-N3_DR	0.8222
	u.3 (R)	-	-
	d.4 (R)	DNA-8-3_DR	0.7634
		SINE2-1 AFC	0.7500
	u.4 (R)	GYPSY68-I_AG	0.8182
Scaf_19	d.1 (R)	MAUI	0.7135
	u.1 (R)	REX1-1_AFC	0.9028
		DNA-8-1_HM	0.8333
	d.2 (R)	REX1-1_AFC	0.9028
		DNA-8-1_HM	0.8333
		DNA-X-7_DR	0.7150
	u.2 (R)	DNA-5-6_DR	0.8022
		EnSpm-6N1_DR	0.7528
		ERV2-9_Pca-I	0.7872
		L2-2_Lme	0.9722
		L2-1_FR1	0.8333
	d.3 (R)	TE-X-4_DR	0.8511
	u.3 (R)	Charlie3_Xt	0.8684
		MAUI	0.7289

		MAUI	0.8302
Scaf_38	d	REX1-4_AFC	0.7812
	u	ERV3-1-I_XT	0.7759
		Helitron-N10_NV	0.8511
		Gypsy-40_Stu-LTR	0.7945
Scaf_40	d (R)	-	-
	u (R)	SINE_FR2	0.7964
Scaf_44	d (R)	L2-8_DR	0.7959
		Chester-1	0.7538
	u (R)	hAT-1_SP	0.8250
		TZF28B	0.8010
		Tc1-1_DR	0.9221
Scaf_58	d (R)	L1PA10	0.7531
	u (R)	-	-
Scaf_61	d	TC1_FR3	0.7449
		SINE_FR2	0.7934
		DIRS-1D-LTR_DR	0.7500
	u	DNA-8-8 DR	0.7407
		EnSpm-8_HM	0.8000
		Chap8_Xt	0.7121
Scaf_86	d (R)	TART_DV	0.8113
	u (R)	MAUI	0.7164
Scaf_104	u	L1-2_Dor	0.8367
		CR1-21_NV	0.7500
		TRE3D	0.7692
	d	-	-
Scaf_114	d (R)	JODY_HC_LTR	0.7692
		Tc1-2_Xt	0.7518
		MARINER_2_DR	0.7600
		SINE2-1 AFC	0.8214
		SINE2-1 AFC	0.8596
	u (R)	Mariner-1_DR	0.7009
Scaf_118	d	ENSPM-6_ZM	0.7639
		Polinton-8_NVi	0.7800
		RTE-1_AFC	0.7818
		piggyback-4_BF	0.7193
	u	SINE2-1_AFC	0.7851
Scaf_136	d	Tc1-5_Xt	0.7305
		Tc1-5_Xt	0.7143
		Tc1-5_Xt	0.7231
	u	Gypsy-614_AA-I	0.7857
		SINE_TE	0.7925
Scaf_143	d (R)	Harbinger-	0.7600
	u (R)	N20_NV	0.8261
		L1-3_SSc	0.8611

		GmGYPSY11_I	0.8367
		CHAPLIN1_FR	0.7917
		Jockey-14_DAn	
Scaf_169	d	MAUI	0.7238
		L2-1_DR	0.7432
		CR1-82_AAe	0.7736
	u	DNA-1-3_DR	0.7677
		CR-74_HM	0.8500
		L2-41_DRe	0.7398
Scaf_181	d(R)	SINE2-1 AFC	0.8049
		SINE2-1 AFC	0.7204
		RR55	0.7188
	u(R)	SINE_FR2	0.8097
		SINE_TE	0.8605
Scaf_182	d	DNA-8-25_DR	0.7561
		Copia-39_Pru-I	0,7778
	u	SINE2-1 AFC	0.8182
Scaf_185	d	Mariner-35_HM	0.7419
		MAUI	0.7412
		Expander	0.7368
	u	RTE-1_AFC	0.7176
		Sat-1_TSy	0.7778
Scaf_197	d	MAUI	0.7500
		SINE_FR2	0.8174
	u	MAUI	0.7394
		L2-1_DR	0.7361
Scaf_201	d (R)	Tc1-1_DR	0.8000
	u (R)	Tc1-1_Xt	0.7562
Scaf_214	d	Tc1-1_FR1	0.7320
		MAUI	0.7797
	u	SINE2-1_AFC	0.8245
		Tc1_FR1	0.8256
		Tc1_FR1	0.8571
Scaf_245	d	Swimmer	0.8000
	u	-	-
Scaf_250	d (R)	CHAPLIN3_FR	0.8125
		hAT-2_Xt	0.8036
		SINE2-1_AFC	0.8819
	u (R)	Kiri_14_AAe	0.8649
		DONG_FR2	0.8626
Scaf_515	d (R)	DNA-3-5_DR	0.7667
		L2-41_DRe	0.7031
		BRIDGE1_TN	0.9143
	u (R)	-	-
Scaf_516	d.2 (R)	-	-
	u.2 (R)	REX1-5_XT	0.7143

		Gypsy-11_RP-I	0.8491
Scaf_834	d	REX1-2_AFC	0.8868
	u	-	-
Scaf_1005	d (R)	SINE_AFC	0.9606
	u (R)	-	-
